# Takotsubo Cardiomyopathy in a Patient with Preexisting Hypertrophic Cardiomyopathy

**DOI:** 10.7759/cureus.3579

**Published:** 2018-11-12

**Authors:** Sherif Elhosseiny, Jonathon Spagnola, Roman Royzman, James Lafferty, Marc Bogin

**Affiliations:** 1 Internal Medicine, Staten Island University Hospital, Staten Island, USA; 2 Cardiology, Staten Island University Hospital, Staten Island, USA

**Keywords:** takotsubo cardiomyopathy, hypertrophic obstructive cardiomyopathy, acute coronary syndrome

## Abstract

Takotsubo cardiomyopathy (TCM) is a condition characterized by transient left ventricular dysfunction and apical ballooning, best seen on an echocardiogram or left ventriculogram. It mimics acute myocardial infarction but without evidence of coronary artery disease on an angiogram. Hypertrophic cardiomyopathy (HCM) is an autosomal dominant heart muscle disease that is significant with hypertrophy of the left ventricle with various morphologies. We hereby report a case of TCM in a male patient with a known history of HCM. The patient’s hemodynamic findings were challenging because the TCM produced an increased left ventricular outflow tract (LVOT) gradient that was previously not seen on his prior echocardiogram or cardiac catheterizations. Assessment and continuous monitoring are warranted in such a rare case. Supportive care afterward with beta blockers, along with echocardiogram surveillance, are the mainstay of management of such a patient.

## Introduction

Takotsubo cardiomyopathy (TCM) is a condition characterized by transient left ventricular dysfunction and apical ballooning, best seen on an echocardiogram or left ventriculogram. On electrocardiogram (ECG), the condition often mimics myocardial infarction; however, these findings occur in the absence of angiographic evidence of obstructive coronary artery disease. Hypertrophic cardiomyopathy (HCM), on the other hand, is a condition seen where cardiomyocytes become regionally hypertrophied, which can result in left ventricular outflow tract (LVOT) obstruction. We hereby report a case of TCM in a male patient with a known history of hypertrophic cardiomyopathy.

## Case presentation

A 67-year-old male with a known past medical history of hypertrophic obstructive cardiomyopathy (HCM) presented to the emergency department (ED) complaining of chest pain lasting for one day. He has a known past medical history of hypertension, dyslipidemia, and coronary heart disease with stents in the left anterior descending artery and left circumflex. However, he was not compliant with his metoprolol and was doing a strenuous activity when he started to feel retrosternal left-sided chest pain, which was pressure-like, non-radiating, and four out of 10 in intensity that was aggravated by lying down. On physical exam, the vital signs were within normal limits, his chest was clear to auscultation, and he had normal S1 and S2 with a harsh systolic murmur best heard over the left sternal border.

**Figure 1:**
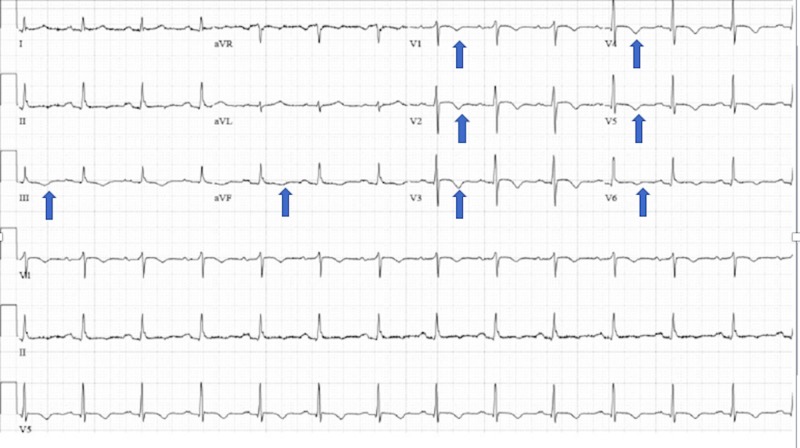
Patient electrocardiogram on admission showing T wave inversions in leads III-aVf and V1-V6

**Figure 2:**
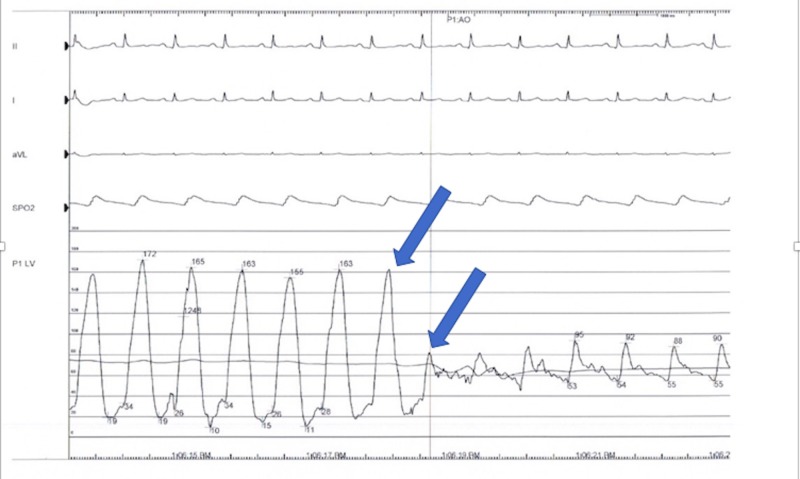
2017 cardiac catheterization showing an intracavitary gradient of 50 mmHg on pullback induced by Takotsubo cardiomyopathy

**Figure 3:**
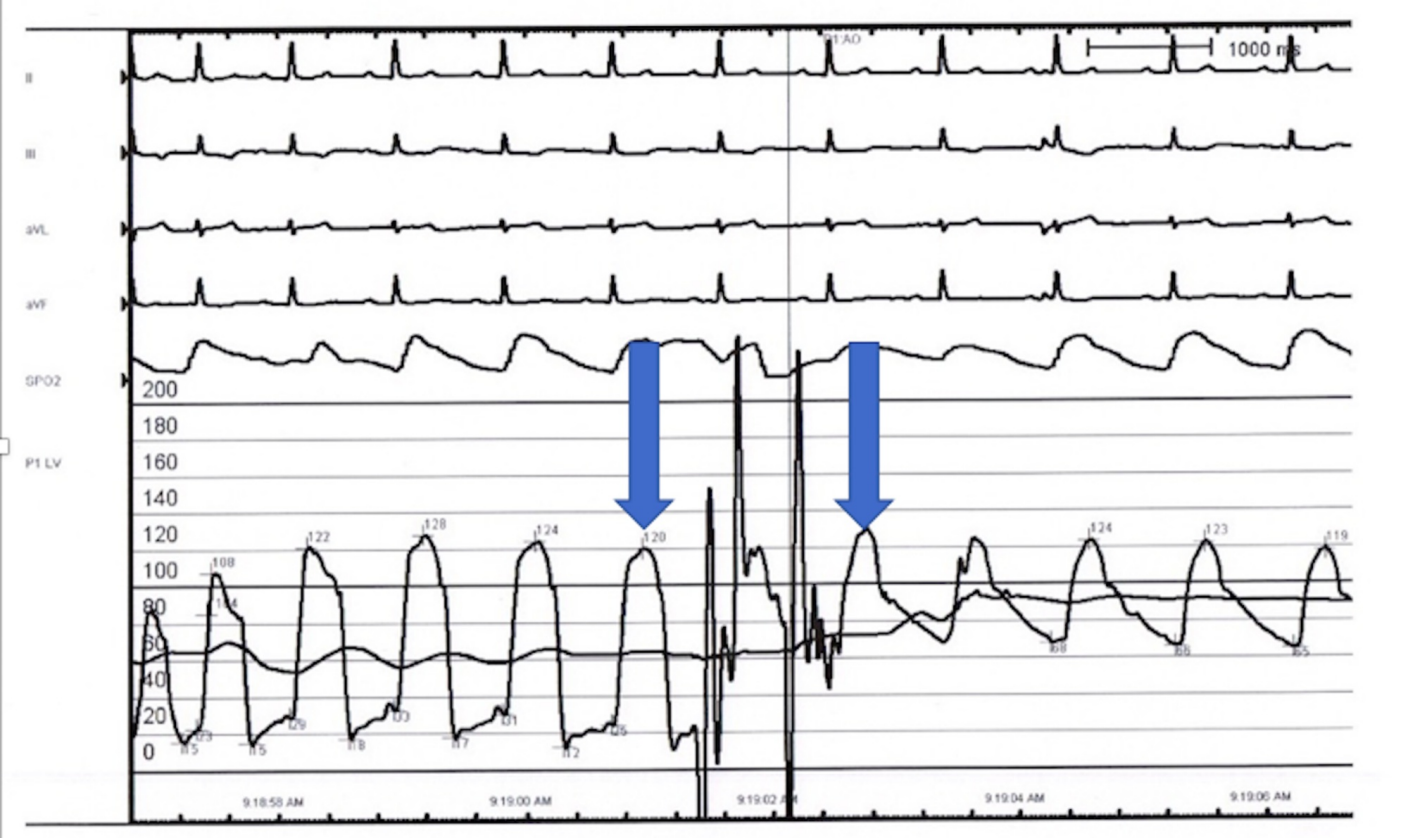
2014 left heart catheterization No gradient seen on pullback across the left ventricular outflow tract (LVOT) and aortic valve.

**Figure 4:**
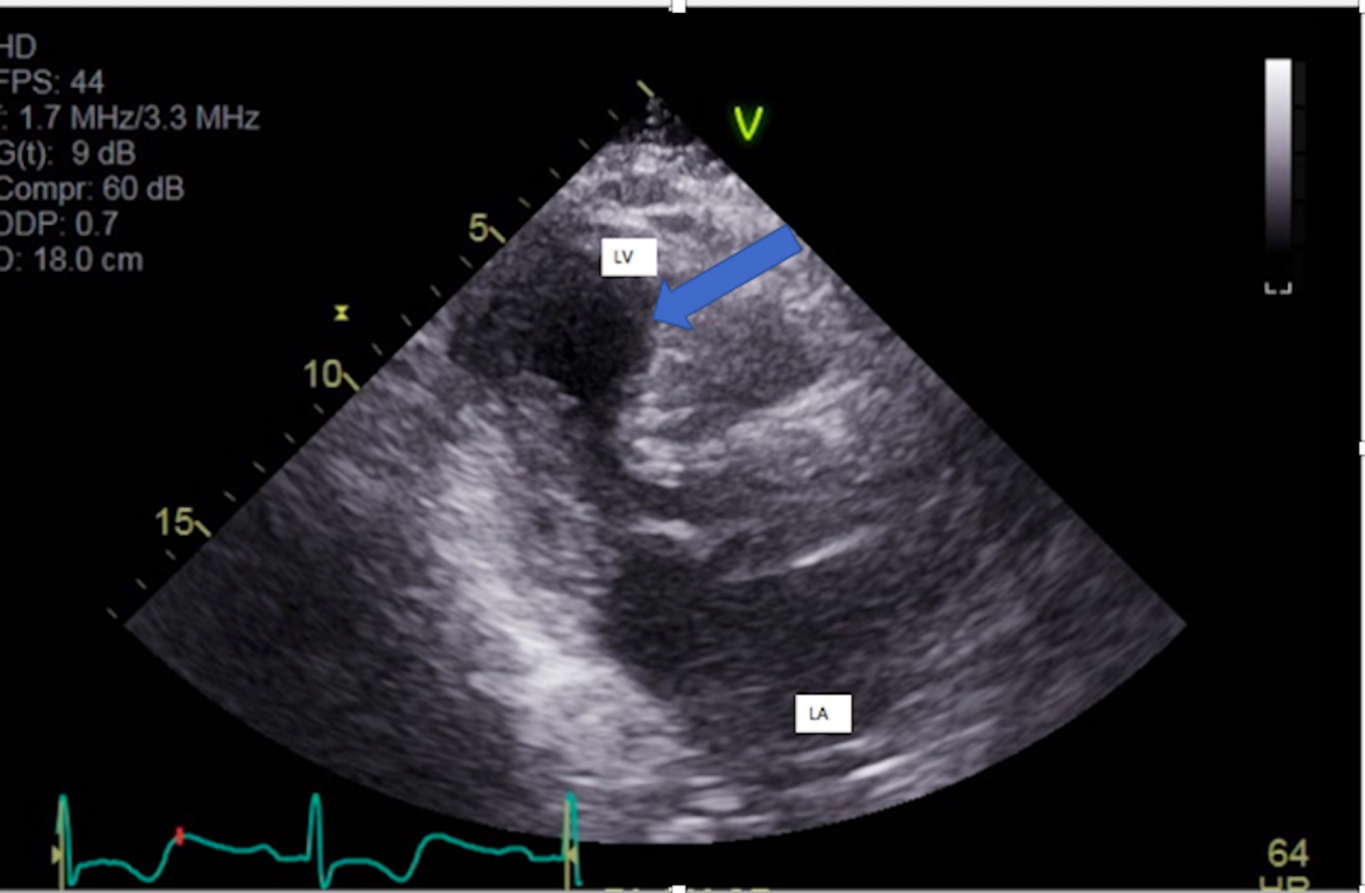
Parasternal long axis view showing apical ballooning due to hypokinesis of the apex, apical interventricular septum, and apical lateral wall LA: left atrium; LV: left ventricle

**Figure 5:**
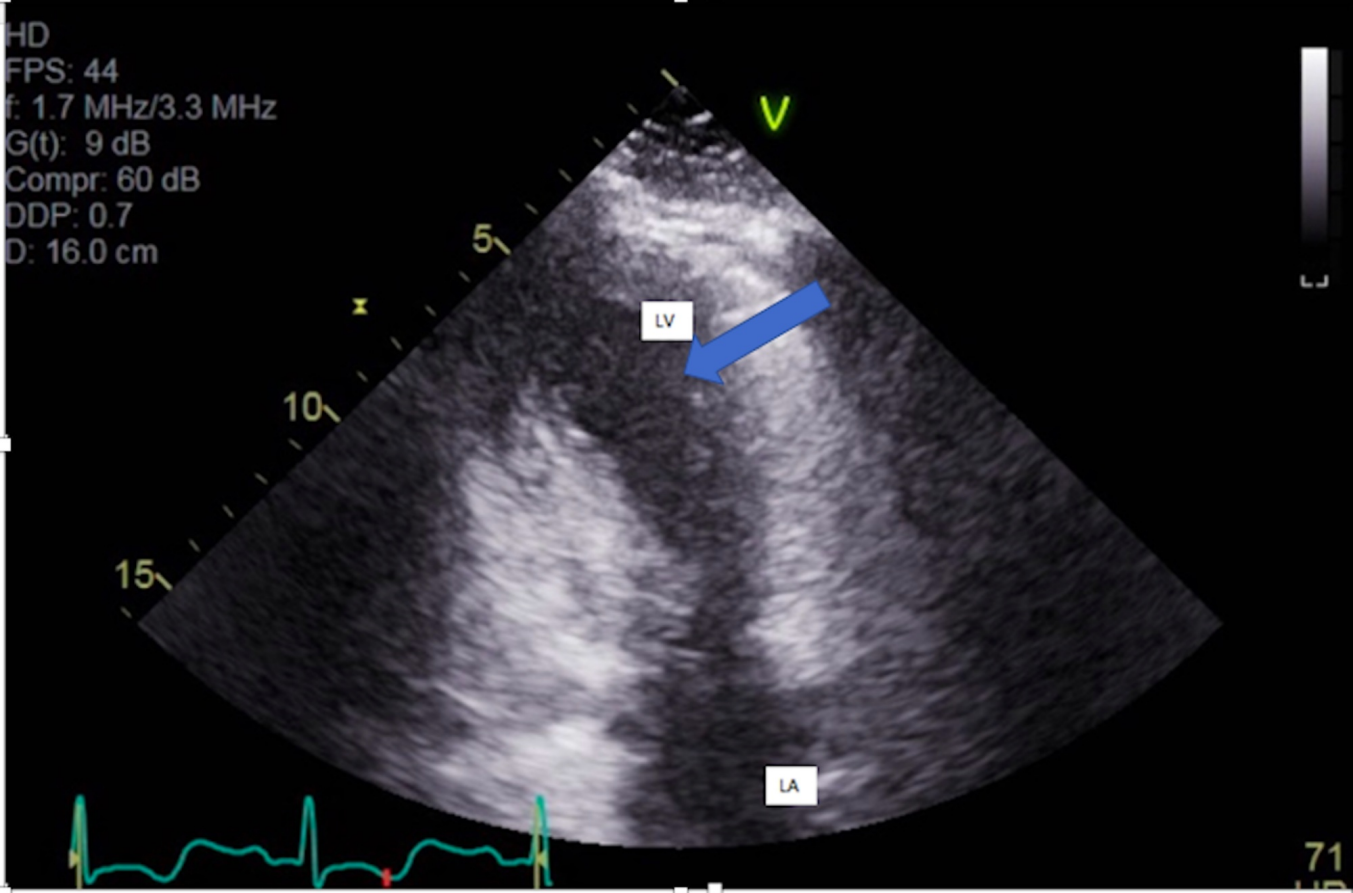
Two chamber views showing apical ballooning due to hypokinesis of the apex, apical interventricular septum, and apical lateral wall LA: left atrium; LV: left ventricle

**Figure 6:**
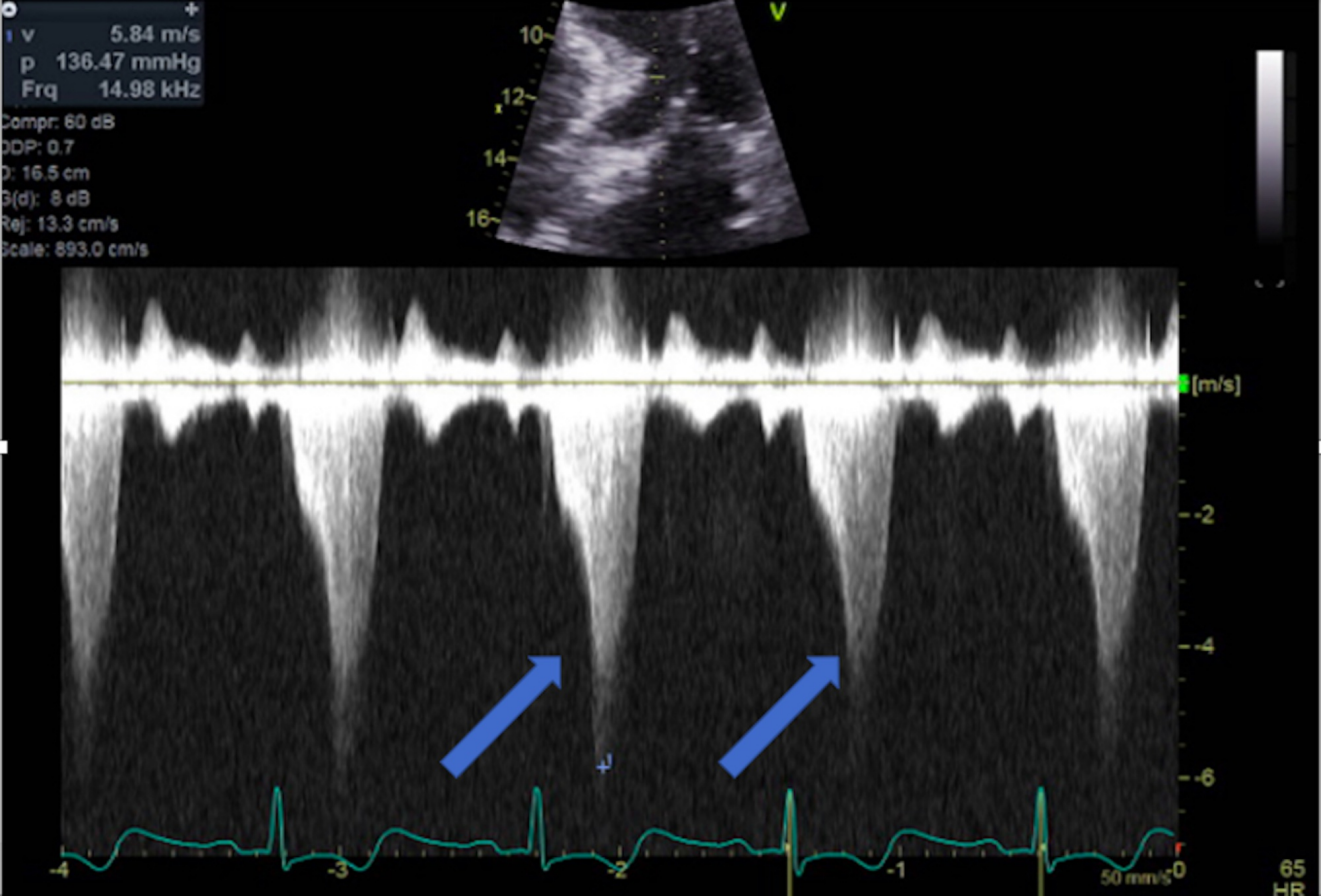
CW Doppler across LVOT at rest showing an increased pressure gradient CW Doppler: continuous Doppler; LVOT: left ventricular outflow tract

**Figure 7:**
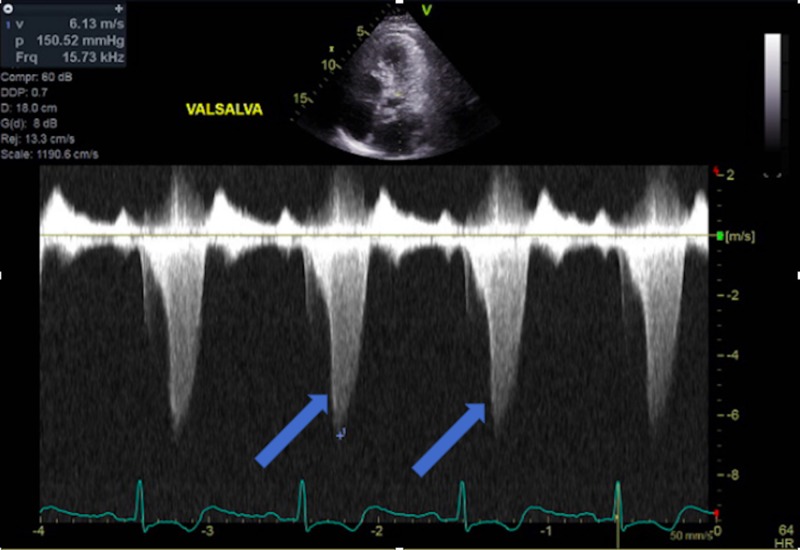
CW Doppler across LVOT with induced Valsalva showing an increased pressure gradient across LVOT CW Doppler: continuous Doppler; LVOT: left ventricular outflow tract

Laboratory evaluation was significant for troponins of 1.5 ng/mL (normal: < 0.05). EKG revealed T wave inversions from V3 to V5 on admission (Figure 1). Upon this hospitalization, he was urgently taken to the cardiac catheterization lab where he was he was found to have non-obstructive coronary artery disease, patent stents, and an intracavitary gradient of 50 mmHg on pullback (Figure 2). This was remarkable as he had not had a left ventricular outflow tract (LVOT) gradient in a previous left heart catheterization three years earlier (Figure 3). An echocardiogram after the catheterization revealed that he had a normal ejection fraction with severe hypokinesis of the apical wall consistent with Takotsubo cardiomyopathy (Figures 4-5). There was a dynamic obstruction during Valsalva in the outflow tract, with a peak velocity of 613 cm/s and an estimated peak gradient of 150 mmHg (Figures 6-7). The patient was started on metoprolol succinate daily, and his condition markedly improved. One month later, a repeat echocardiogram showed a normal ejection fraction with a resolution of the apical hypokinesis and an exercise-induced left ventricular outflow tract gradient (LVOT) of 80 mm Hg, consistent with hypertrophic cardiomyopathy. This denotes that the TCM had worsened the patient's LVOT obstruction, and upon resolution of it, the patient's hemodynamics returned back to baseline. The patient was asymptomatic and had no complaints.

## Discussion

Takotsubo cardiomyopathy (TCM) was first described in Japan in 1990 as a syndrome characterized by transient regional systolic dysfunction of the left ventricle (LV) similar to a myocardial infarction but with the absence of angiographic evidence of obstructive coronary artery disease. The term Takotsubo comes from the Japanese name for an octopus trap, which is similar in shape to that of systolic apical ballooning of the LV in the most common and typical form of the disorder [1-4]. Takotsubo cardiomyopathy is also referred to as an apical ballooning syndrome, stress-induced cardiomyopathy, or broken heart syndrome. TCM occurs predominantly in older adults and is more common in women than men [2-3, 5]. The pathogenesis behind TCM is not well understood. However, it is most frequently triggered by physical or emotional stress, i.e., death, abuse, devastating financial or gambling losses). This suggests that this disorder may be caused by diffuse catecholamine-induced microvascular spasm or a dysfunction leading to myocardial stunning and/or necrosis [3, 6-8]. Patients typically present with substernal chest pain. It has also been found that it is complicated with LVOT obstruction and hypotension. In one retrospective study of 3,272 patients presenting with an acute coronary syndrome, 32 patients were ultimately diagnosed with TCM (1% of patients). Of these, 20% (n = 6) had LVOT obstruction identified or confirmed by transthoracic echocardiography (TTE) [9]. Some patients develop complications, such as heart failure, tachyarrhythmias, bradyarrhythmias, sudden cardiac arrest, and significant mitral regurgitation [2-3, 5]. The recommended approach for a hypotensive patient with TCM and LVOT obstruction is usually the suppression of contractility with β-blocker use. In contrast to patients with hypotension secondary to heart failure, hypotension due to LVOT obstruction should not be treated with inotropic agents as this will worsen the degree of the obstruction [4].

Hypertrophic cardiomyopathy (HCM) is an autosomal dominant heart muscle disease affecting one in every 500 adults. HCM presents with hypertrophy of the left ventricle with various morphologies [10-12] and is most commonly caused by a mutation in one of several sarcomere genes that is responsible for muscle formation [13-15]. Echocardiography is a cornerstone of diagnosing patients with HCM [16]. Patients with HCM have varying presentations; while many patients are asymptomatic, other patients will present with dyspnea on exertion, atypical or anginal chest pain, and arrhythmias leading to pre-syncope, syncope, and/or sudden cardiac death [10-12, 17-18]. As previously stated, TCM can be complicated with LVOT obstruction; however, a very limited number of cases have described TCM in patients with known HCM. Some of these patients were previously diagnosed with HCM, while others were only diagnosed with HCM when they presented with TCM [19-20]. Our patient is challenging and unique due to his history; a non-compliant 67-year-old male who was previously diagnosed with stress-induced LVOT obstruction and HCM. He had a previous catheterization with stent insertion in which there was no LVOT pressure gradient (Figure 3). However, when the patient presented with TCM, his catheterization showed an LVOT pressure gradient of 50 mmHg. An echocardiogram showed a worsening LVOT peak gradient of 150 mmHg (Figures 6-7). Upon follow-up after one month of medical therapy, his LVOT gradient returned back to its baseline and his exercise LVOT gradient was 80 mmHg. This denotes that this patient’s TCM worsened his HCM from a hemodynamic standpoint, and once the patient recovered from TCM, his gradient improved on echo. Therefore, TCM can worsen LVOT obstruction in patients with pre-existing HCM and can lead to dangerous symptomatology.

Management in such patient groups can be challenging in the acute setting, especially when there is a severe LVOT gradient leading to low cardiac output. The mainstay of treatment initially is supportive care with beta blockers. Reversal of the TCM is expected with time and appropriate medical therapy. Careful surveillance with echocardiography and stress echocardiography is required to monitor disease progression in such patients. These patients should have close follow-up with a structural cardiology team in case medications are not sufficient enough to control symptoms.

## Conclusions

Takotsubo cardiomyopathy (TCM) is a challenging condition that mimics the presentation of an acute coronary syndrome. The diagnosis is usually established with negative cardiac catheterization and typical findings on echocardiogram. We hereby report a unique case of TCM in a male patient with a known history of HCM. The patient’s hemodynamic findings here are of particular interest because the TCM produced an increased LVOT gradient that was previously not seen on his prior echo or cardiac catheterizations. The combination of the two pathologies can lead to low cardiac output, worsening shortness of breath, syncope, and exertional dyspnea. Careful initial evaluation and continuous monitoring are warranted in such rare cases. Supportive care afterward with beta blockers, along with echocardiogram surveillance, are the mainstay of management. A structural cardiology specialist should be involved in the ongoing care of patients to monitor for resolution of symptoms, lowering of the LVOT gradient, and to determine if any procedural treatment for HCM is indeed necessary.
